# Rationale and Development of Tavapadon, a D1/D5-Selective Partial Dopamine Agonist for the Treatment of Parkinson’s Disease

**DOI:** 10.2174/1871527322666230331121028

**Published:** 2024-01-10

**Authors:** Erwan Bezard, David Gray, Rouba Kozak, Matthew Leoni, Cari Combs, Sridhar Duvvuri

**Affiliations:** 1 Université de Bordeaux, CNRS Institut des Maladies Neurodégénératives, UMR 5293, Bordeaux, France;; 2 Motac Neuroscience, Manchester, United Kingdom;; 3 Inscopix, Mountain View, CA, USA;; 4 Novartis, Cambridge, MA, USA;; 5 Cerevel Therapeutics, Boston, MA, USA

**Keywords:** Dopamine receptors, D1/D5 partial dopamine agonist, D1 agonist, direct pathway, indirect pathway, motor symptoms, Parkinson’s disease, tavapadon

## Abstract

Currently, available therapeutics for the treatment of Parkinson’s disease (PD) fail to provide sustained and predictable relief from motor symptoms without significant risk of adverse events (AEs). While dopaminergic agents, particularly levodopa, may initially provide strong motor control, this efficacy can vary with disease progression. Patients may suffer from motor fluctuations, including sudden and unpredictable drop-offs in efficacy. Dopamine agonists (DAs) are often prescribed during early-stage PD with the expectation they will delay the development of levodopa-associated complications, but currently available DAs are less effective than levodopa for the treatment of motor symptoms. Furthermore, both levodopa and DAs are associated with a significant risk of AEs, many of which can be linked to strong, repeated stimulation of D2/D3 dopamine receptors. Targeting D1/D5 dopamine receptors has been hypothesized to produce strong motor benefits with a reduced risk of D2/D3-related AEs, but the development of D1-selective agonists has been previously hindered by intolerable cardiovascular AEs and poor pharmacokinetic properties. There is therefore an unmet need in PD treatment for therapeutics that provide sustained and predictable efficacy, with strong relief from motor symptoms and reduced risk of AEs. Partial agonism at D1/D5 has shown promise for providing relief from motor symptoms, potentially without the AEs associated with D2/D3-selective DAs and full D1/D5-selective DAs. Tavapadon is a novel oral partial agonist that is highly selective at D1/D5 receptors and could meet these criteria. This review summarizes currently available evidence of tavapadon’s therapeutic potential for the treatment of early through advanced PD.

## 1 INTRODUCTION

Parkinson’s disease (PD) is a progressive neurodegenerative disease characterized by deficits in motor control arising from the loss of dopaminergic neurons in the substantia nigra pars compacta and subsequent depletion of dopamine in motor regions of the striatum [1-4]. Current pharmacological treatment approaches span several different drug classes, including dopamine precursors (*e.g.* levodopa), dopamine agonists (DAs; *e.g.* pramipexole, ropinirole, rotigotine, apomorphine), monoamine oxidase B (MAO-B) inhibitors (*e.g.* rasagiline, selegeline, safinamide), anticholinergics (*e.g.* benztropine), catechol-O-methyl transferase (COMT) inhibitors (*e.g.,* entacapone, opicapone), N-methyl-D-aspartate (NMDA)-type glutamate receptor antagonists (*e.g.* amantadine), and A_2A_ antagonists (*e.g.* istradefylline) [3, 5-7]. Additional nonpharmacological treatments, such as deep brain stimulation, are also available [5]. Finally, disease-modifying therapies are under investigation with the aim to slow or stop the progression of PD [8]. Many of the investigational therapies in various stages of clinical and preclinical development target the pathological build-up of α-synuclein, a protein found in the presynaptic membrane that has been implicated in the degeneration of dopamine neurons in PD [8, 9]. Other approaches, including calcium channel blockers, anti-inflammatory therapies, treatments aimed at increasing levels of the antioxidant glutathione, and therapies targeting specific monogenetic forms of PD, may also have the potential to slow PD-related neurodegeneration [10]. While these treatments may hold promise for the future treatment of PD, no disease-modifying therapies are approved for PD to date and effective symptomatic therapies continue to be needed for the treatment of PD [8, 11].

Symptom improvements with current pharmacological therapies are often transient and unpredictable following prolonged treatment; for example, patients may report periodic fluctuations in treatment efficacy, a phenomenon referred to as “ON/OFF” fluctuations [4, 12]. These fluctuations require dose escalation and treatment switching to manage motor efficacy while balancing the adverse events (AEs) associated with many anti-PD therapeutics [13, 14]. Recent American Academy of Neurology (AAN) guidelines recommend levodopa as the preferred therapy for initial treatment of early-stage PD because of its ability to produce greater motor benefits than currently available DAs and MAO-B inhibitors and lessen the risk of AEs linked to these other agents, such as excessive daytime sleepiness (EDS) and impulse control disorders (ICDs) [5, 6, 15-17]. However, due to its short half-life, levodopa is administered multiple times a day [18, 19]. As the disease progresses, levodopa is associated with unpredictable fluctuations in efficacy (“ON/OFF” periods), as well as a decrease in the duration of improvements after administration [12, 19]. These reductions in efficacy are partially due to the short half-life of levodopa but also have been linked to the desensitization of dopamine receptors following prolonged treatment and during disease progression [18-20]. For advanced disease, adjunctive therapy with DAs and lower dose levodopa may be recommended to allow for motor control without the risk of troublesome AEs associated with higher doses of levodopa, but clinical evidence for the benefits of combined treatment with currently approved DAs remains unclear [5]. Therefore, novel therapeutics that deliver sustained and predictable motor improvements without significant motor fluctuations are needed.

**Fig. (1):**
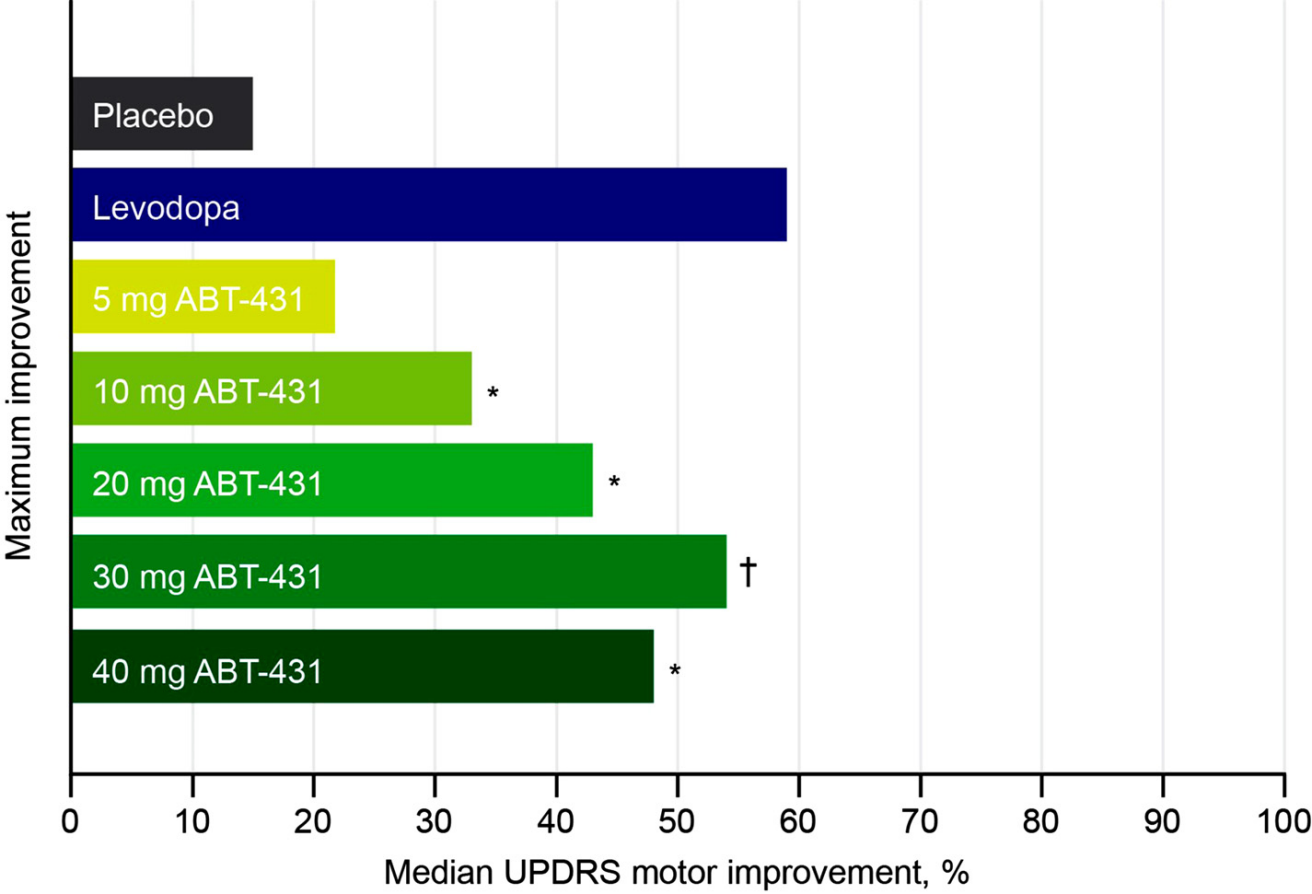
Maximum improvement in UPDRS motor score following treatment with ABT-431 or levodopa in patients with PD [39]. *P < 0.05; ^†^*P* < 0.005, compared with placebo. PD, Parkinson’s disease; UPDRS, Unified Parkinson’s Disease Rating Scale.

Currently available dopaminergic treatments act by either increasing dopamine levels (*e.g.* levodopa, COMT, and MAO-B inhibitors) or directly activating the dopamine receptors (*e.g.* DAs) in the striatum [3]. Dopamine receptor subtypes can be grouped into two specific receptor families, the D1 and D5 receptors, and D2, D3, and D4 receptors [21]. The degree of motor control observed with current pharmacological therapies for PD may partly depend on which dopamine receptor subtypes, and consequently, which motor pathways, are activated. Within the striatum specifically, dopamine acts on two distinct receptor populations, primarily D1/D5 and D2/D3 receptors, which differ in the neuronal populations on which they are expressed and in the G-proteins to which they are coupled (G_s_/G_olf_ and G_o_/G_i_ respectively) [21-24]. D1/D5 receptors coupled to stimulatory G_s_/G_olf_ are predominantly expressed on medium spiny neurons (MSNs) of the direct pathway, which projects to striatal nuclei that directly regulate motor output [21]. Conversely, the D2/D3 receptors coupled to inhibitory G_i_/G_o_ are expressed primarily on indirect pathway MSNs, which regulate motor output *via* multisynaptic connections to the basal ganglia [21, 22]. This segregation within the striatum is largely maintained, however, subsets of D3 receptors may also be expressed on MSNs of the direct pathway and presynaptically on dopamine terminals, while some D5 receptors may be expressed on MSNs of the indirect pathway and cholinergic interneurons [24, 25]. Activation of direct pathway MSNs facilitates movement through disinhibition of the thalamus, while activation of the indirect pathway acts as a brake on motor output [26]. Dopamine, therefore, promotes movement by acting on D1/D5 receptors to activate direct pathway MSNs and also by acting on D2/D3 receptors to inhibit indirect pathway MSNs in order to release the inhibitory brake on motor output [27, 28]. Together, these two parallel circuits coordinate purposeful motor control in the healthy brain [26]. The progressive loss of dopamine signaling in PD leads to disruptions in the balance of direct and indirect pathway activation and subsequent dysregulation of striatal outputs [29]. This is further complicated by receptor differences in the affinity for dopamine [30], which under hypodopaminergic conditions such as those in PD, may lead to further imbalance *via* preferential activation of D2/D3-expressing neurons of the indirect pathway. As a dopamine precursor, levodopa leads to the activation of all dopamine receptor subtypes, in addition to targeting extrastriatal dopamine pathways that are not degenerated in PD [31, 32]. Its ability to modulate both the direct and indirect pathways may be a potential reason why levodopa is superior to DAs at improving motor control [5, 16, 33]. Most currently used DAs preferentially target D2/D3 dopamine receptors [3], which may lead to selective effects on indirect pathway neurons and contribute to the less robust motor effect compared with levodopa [16]. It follows that selective activation of the direct pathway may be an alternative and promising approach for the improvement of motor control in PD. In preclinical models of PD, selective activation of the direct pathway improves motor control and D1/D5 MSNs show enhanced responsiveness to activation of D1/D5 receptors by levodopa or a D1/D5-selective DA, SKF-81297 [34-36]. Further, D1/D5-selective (but not D2/D3-selective DAs) have been shown to provide relief of PD motor symptoms even in animals with progressive neurodegeneration that are unresponsive to levodopa [33, 37, 38]. The significant potential of D1/D5 receptor activation for treating PD symptoms, which was first shown in two previous clinical studies of the D1/D5-selective DA ABT-431, found that targeting D1/D5 dopamine receptors can produce robust motor effects equivalent to those observed after administration of levodopa (Fig. **1**) [31, 39, 40]. However, motor improvements following treatment with ABT-431 were also associated with dyskinesia that was equivalent to levodopa, and ABT-431 was not further investigated for treatment of PD [31]. Collectively, this evidence suggests that precisely tuned D1/D5-selective agonism may be a promising approach for improved motor control.

The safety and tolerability of the currently available dopaminergic PD therapies may also be influenced by their dopamine receptor subtype selectivity. Levodopa and DAs activate receptors not only within the striatum but also throughout extrastriatal brain regions, in which D1/D5 and D2/D3 receptors show differential patterns of expression [3, 11, 41]. While both D1/D5 and D2/D3 dopamine receptors are expressed in the cortex, dopaminergic projection nuclei, and dorsal and ventral striatum, D2/D3 receptors are also found throughout additional extrastriatal regions, including the hypothalamus and hindbrain, which means that activation of D2/D3 receptors may result in other effects outside of motor activation [41, 42]. In particular, activation of D2/D3 receptors in the hindbrain has been linked to sleep disruptions [15]. Some AEs observed with currently available D2/D3-selective DAs, including ICDs and cognitive AEs (*e.g.* confusion, hallucinations) are consistent with preclinical and other research linking D2/D3 dopamine receptors expressed within mesolimbic and corticostriatal dopamine pathways to the regulation of impulsivity, reward seeking, and cognition, and may result from extrastriatal, tonic activation of D2/D3 receptors [11, 12, 43, 44]. As a nonselective dopaminergic therapy that increases dopamine signaling in brain regions that are not subject to degeneration in PD, levodopa can produce similar effects, although to a lesser degree [12, 45]. Additionally, the short half-life and full agonism of levodopa promote overall sensitization of dopamine circuitry and upregulation of D1 and D3 dopamine receptors [12, 46], which may explain the higher rate of dyskinesias observed with levodopa when compared with other PD treatments [47]. The more restricted expression pattern of D1/D5 receptors may reduce the risk of these AEs, and activation of D1/D5 receptors in the cortex may also have therapeutic potential for the treatment of nonmotor symptoms in PD, including improvements in cognition and motivation [48]. At the same time, activation of extrastriatal D1/D5 receptors, especially those outside of the central nervous system, can also produce undesired effects [49]. Indeed, previous development of D1/D5-selective DAs has been restricted by their association with cardiovascular and dyskinetic AEs, poor tolerability, and suboptimal bioavailability [31, 39, 49-51]. However, some of these AEs may again be related to full agonism of D1/D5 dopamine receptors [51]. Therefore, it is possible that these AEs may be largely avoided with partial D1/D5-selective agonism.

Tavapadon, a highly selective partial agonist at D1 and D5 receptors, is being investigated for early through advanced PD [52]. Preclinical and early clinical evidence suggests that tavapadon offers the potential to provide robust sustained and predictable motor control *via* selective activation of direct pathways MSNs, coupled with a reduced risk of AEs observed with prior DAs due to its D1/D5 selectivity and partial agonist properties [50]. In the current review, we provide an overview of the evidence generated to date that together supports the therapeutic potential of tavapadon for the treatment of PD.

## 2 PRECLINICAL AND PHARMACOLOGICAL EVIDENCE SUPPORTING D1/D5 PARTIAL AGONISM FOR THE TREATMENT OF PD

### 2.1 Tavapadon Receptor Selectivity and Partial Agonism Properties

Tavapadon is a highly selective partial agonist at D1 and D5 dopamine receptors, [52] with little to no functional activity at D2, D3, or D4 receptors *in vitro* (unpublished data). Assays measuring the displacement of radioligand binding in cell lines expressing recombinant human dopamine receptors have shown that tavapadon has a high affinity for both D1 (*K*_i_ = 9 nM) and D5 (*K*_i_ = 13 nM) (unpublished data). Conversely, tavapadon had a low affinity at D2 (*K*_i_ ≥ 6210 nM), D3 (*K*_i_ ≥ 6720 nM), and D4 (*K*_i_ ≥ 4870 nM) (unpublished data).

The partial agonism profile of tavapadon at D1/D5 receptors is central to its development, as prior *in vitro* evidence suggests that partial agonism of dopamine receptors can produce biased activation of specific intracellular signaling pathways and may provide symptom relief while ameliorating AEs associated with full agonism [12, 20, 33]. Specifically, binding of dopamine-like, catechol-based full D1/D5 DAs induces activation of D1/D5 dopamine receptors *via* interactions with amine and aspartate residues on transmembrane domain 3 and serine residues on transmembrane domain 5 of the D1 dopamine receptor [20]. Prolonged receptor activation then leads to the intracellular association of β-arrestin and subsequent receptor endocytosis, ultimately leading to desensitization of the receptor [20]. Computational modeling of binding interactions between D1 dopamine receptors and a non-catechol-based D1/D5-selective agonist similar to tavapadon (PF-6142) showed no direct binding to transmembrane 3 amine and aspartate residues and reduced association with serine residues in transmembrane 5, instead binding to extracellular domains of the receptor [20]. Live-cell fluorescence imaging experiments demonstrated that PF-6142 was able to activate functional downstream signaling cascades with reduced recruitment of β-arrestin when compared to dopamine, largely preventing receptor desensitization [20]. This lack of functional receptor desensitization can be extrapolated to a reduced risk of behavioral tolerance *in vivo* following repeated drug administration [20], which is important for the sustained therapeutic benefit [53, 54]. *In vitro* assays of functional activity have confirmed that tavapadon acts as a partial agonist by binding at D1 and D5 receptors, corresponding to 65% and 81% of dopamine’s intrinsic activity, respectively, and inducing functional receptor activation, with half-maximal effective concentration (EC_50_) values of 19 nM and 17 nM (unpublished data).

*In vivo* preclinical work has provided further insight into the binding kinetics of tavapadon. Blood plasma levels were used to determine the unbound brain exposure profile in a nonhuman primate model of PD [33]. The peak receptor occupancy at D1 receptors was estimated to be 48% following the administration of 0.1 mg/kg of tavapadon, a dose that led to motor improvements comparable to those seen following levodopa administration [33]. Evidence from this study also suggests that lower doses of tavapadon that may have therapeutic potential as adjunctive therapy in combination with levodopa produce dose-proportional occupancy of D1/D5 receptors.

### 2.2 Preclinical Evidence for Motor Control With D1/D5-Selective Agonism

**Fig. (2):**
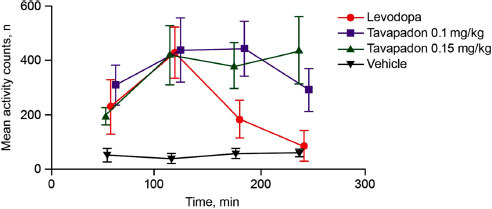
Comparison of tavapadon and levodopa on motor activity in an MPTP-model of PD in nonhuman primates. Higher doses of tavapadon (0.1 and 0.15 mg/kg) led to an improved duration of efficacy versus levodopa. MPTP, 1-methyl-4-phenyl-1,2,3,6-tetrahydropyridine; PD, Parkinson’s disease. Adapted with permission from Young D, Popiolek M, Trapa P, *et al.* ACS Chem Neurosci 2020; 11(4): 560-6 [33]. ^©^Copyright 2020 American Chemical Society.

Preclinical research in rodents and nonhuman primates supports the potential for motor control with tavapadon in PD. In a preclinical study using the 1-methyl-4-phenyl-1,2,3,6-tetrahydropyridine (MPTP) model of PD in nonhuman primates, tavapadon led to improvements in motor function on par with those seen following levodopa administration (Fig. **2**) [33]. Motor improvements following tavapadon administration were sustained for more than twice as long as those seen following treatment with levodopa, supporting potential sustained motor benefit with tavapadon (Fig. **2**) [33]. Tavapadon also reduced motor symptoms when administered in combination with lower doses of levodopa [33]. These results parallel earlier findings demonstrating that full D1/D5-selective DAs reduced motor symptoms of PD in preclinical models [55]. Indeed, administration of the full D1/D5-selective DA dihydrexidine [56] led to improvements in motor symptoms in a primate model of severe MPTP-induced PD, even when subject animals were unresponsive to levodopa [37]. Similar effects were not seen following the administration of the D2/D3-selective agonist bromocriptine [37]. Tavapadon may also provide advantages over previously investigated full D1/D5-selective DAs (*e.g.* A-86929 and its precursor ABT-431) not only in terms of its sustained motor benefit but also in the reduced risk of troublesome dyskinesias [31, 33, 55]. The preclinical study of tavapadon was conducted using nonhuman primates with MPTP-induced PD that had previously developed dyskinesias in response to long-term treatment with levodopa [33]. Even so, administration of tavapadon either alone or in combination with levodopa resulted in strong control of motor symptoms accompanied by reduced dyskinesias [33]. This evidence for tavapadon indicates the potential for robust motor control, with a lower likelihood of motor fluctuations or dyskinesias [33].

### 2.3 Preclinical and Other Evidence Regarding Safety Profiles of D1/D5-Selective *vs.* D2/D3-Selective Agonism

A commonly reported AE associated with currently used D2/D3 DAs is the risk of ICDs [43, 45]. ICDs are reported in 17% of patients with PD taking D2/D3 DAs, a significantly greater rate of ICDs than observed with patients with PD not taking D2/D3 DAs (7%) [43]. Although clinical research suggests that D2/D3 receptors are linked to the development of ICDs following treatment with D2/D3 DAs, it has not yet been able to parse the relative contributions of dopamine receptor activation, PD-related neurodegeneration, and underlying predisposition to ICDs [43, 57, 58]. Preclinical work may offer some insight, as a wide body of preclinical evidence suggests that the risk of disinhibition and ICDs following the administration of current DAs is likely driven by the activation of D2/D3 dopamine receptors [58]. For example, both acute and chronic administration of D2/D3-selective agonists induces compulsive phenotypes in rats. [59, 60]. Long-term administration of the D2/D3-selective agonists ropinirole or pramipexole results in a biased choice of large, uncertain rewards and impairs risk/ reward learning, which are classic characteristics of ICDs [57, 61, 62]. Importantly, the effects of these D2/D3-selective agonists on impulsivity in preclinical studies did not differ in PD animals *vs.* controls [57, 61]. Further, these compulsive behaviors stopped following cessation of D2/D3 DA administration, consistent with reports in patients with PD that ICDs often resolve following discontinuation of treatment with D2/D3 DAs [58, 61]. These findings demonstrate that the development of ICDs in patients with PD could be a direct consequence of D2/D3 DA treatment [57].

Although striatal circuitry has been implicated in the pathogenesis of ICDs, the effects of D2/D3-selective DAs on ICDs may also be driven by extrastriatal effects [44, 58]. Selective activation of D2/D3 dopamine receptors, but not D1/D5 dopamine receptors, within the medial prefrontal cortex impaired risk-based decision-making, similar to the effects seen following systemic administration of D2/D3-selective DAs [57, 61, 63]. Similar effects were observed in the ventral striatum, where amphetamine-induced increases in impulsivity were dependent on D2/D3, but not D1/D5 dopamine receptors [64]. While there was no effect of activating D1/D5 receptors in the orbitofrontal cortex, administration of the D2/D3-selective DA quinpirole to the orbitofrontal cortex reduced impulsive responding [65]. These results together suggest that D2/D3 dopamine receptors throughout corticostriatal and corticolimbic pathways play a complex role in the regulation of impulsive behaviors, while D1/D5 dopamine receptors may not [58].

While D1/D5 dopamine receptors have not been implicated in the development of ICDs, this system has been widely studied in relation to drug-seeking behaviors [58, 66]. In some patients with PD, the use of dopaminergic therapies can become compulsive, a phenomenon referred to as dopamine dysregulation syndrome [43]. These patients exhibit many behaviors characteristic of drug addiction, including escalation of drug use, avoidance of OFF periods associated with drug withdrawal, and persistence of drug use in the face of negative consequences [46, 67]. The majority of patients who develop dopamine dysregulation therapy syndrome are treated with levodopa (88% of cases), although this profile may also develop with concurrent or monotherapy use of DAs (55% of cases) [68]. It is therefore challenging to tease apart any disproportionate, relative contributions of specific receptor systems to the development of this behavior from clinical literature alone [67].

In preclinical literature, D1/D5-selective agonists have reliably reduced drug-seeking behaviors [66]. Application of D1/D5-selective agonists reduces relapsing behaviors in rodents and nonhuman primates [66, 69-71]. These results have been extended to effects on ethanol, nicotine, and food intake, indicating that D1/D5 receptors are broadly involved in reward-seeking behaviors [72, 73]. Importantly, these effects are maintained with the administration of partial D1/D5-selective DAs [69, 73], suggesting that tavapadon may share some of these characteristics. Conversely, preclinical data suggest that D2/D3-selective agonists enhance relapse to drug seeking and are in some cases administered on their own [70, 71, 74]. The role of D1/D5 *vs.* D2/D3 dopamine receptors in mediating reward-seeking behaviors has been replicated in preclinical models of PD, and degeneration of the dopamine system may actually make animals more sensitive to the effects of D2/D3-selective agonists [74-77]. While the D1/D5-selective agonist ABT-431 did not reduce drug-seeking behaviors in a trial measuring the choice of cocaine in 9 experienced cocaine smokers, it did reduce self-reported subjective effects of cocaine, indicating that some of these preclinical findings are replicated in clinical populations [78]. Although the evidence described above is indirect, these data suggest that D1/D5-selective DAs may have less liability for misuse than currently available dopamine replacement therapies, such as levodopa or D2/D3-selective DAs [77].

## 3 CLINICAL EVIDENCE FOR D1/D5-SELECTIVE PARTIAL AGONISM IN PARKINSON’S DISEASE

The clinical potential of tavapadon has been explored in phase 1 and 2 trials (Table **1**) [50, 52]. The safety, tolerability, pharmacokinetic (PK), and pharmacodynamic profiles of tavapadon were assessed in single ascending-dose (SAD; N=18) and multiple-ascending-dose (MAD; N=50) trials [50]. In both trials, the PK profile of tavapadon was evaluated in patients with a clinical diagnosis of idiopathic PD [50]. Peak plasma concentrations were achieved 3-4 hours after administration of a single oral dose under fasted conditions [50]. Similar PK patterns were seen following 22 days of titrated administration and when dosing followed consumption of a high-fat meal, suggesting that food may not have a clinically relevant impact on the rate or extent of absorption of tavapadon [50, 79]. The half-life of tavapadon is approximately 24 hours [80], which is substantially longer than that of levodopa or standard formulations of many D2/D3-selective DAs [18, 81, 82]. The extended half-life and predictable bioavailability of tavapadon enable once-daily dosing and may offer improvements for sustained motor control while reducing the likelihood of dyskinesias [12, 50].

In addition to the PK profile characterization, both the SAD and MAD trials provided initial pharmacodynamic assessments of motor control for tavapadon [50]. In the SAD trial, the administration of tavapadon resulted in rapid improvements in motor control [50]. On the first day of treatment with 9.0 mg of tavapadon, patients showed a significant reduction in motor symptoms compared with placebo starting one hour after drug administration, as measured by the Movement Disorder Society-Unified Parkinson’s Disease Rating Scale Part III (MDS-UPDRS-III) [50]. Importantly, these improvements were sustained for at least 12 hours after treatment, providing further support for a once-daily dosing regimen [50]. There was a similar reduction in MDS-UPDRS-III scores that started one hour after administration of 3.0 mg of tavapadon and lasted 12 hours, although this was not significantly different from the placebo [50]. In the open-label MAD trial, motor effects were evaluated on day 22, following titrated administration of multiple oral doses of tavapadon [50]. Two of the four cohorts included in the MAD trial showed a sustained reduction in MDS-UPDRS-III scores at this time point [50].

These SAD and MAD trials also provided initial support that tavapadon may be well tolerated with an acceptable AE profile in patients with PD [50]. In contrast to the large drop in blood pressure reported in previous clinical trials of full D1-selective agonists (*e.g.* ABT-431, dihydrexidine) [31, 39, 51], tavapadon produced only modest dose-related changes in cardiovascular measures that were not clinically meaningful [50]. There were no deaths, serious or severe AEs, discontinuations due to AEs, or dose reductions in any of the patients in the SAD trial [50]. In the MAD trial, the majority of AEs were mild or moderate, with the exception of six severe AEs, and 11 patients discontinued treatment due to AEs; the majority (7/11) of these patients were from the cohort that received the 25 mg once-daily dose of tavapadon [50], which is higher than the doses currently being explored for efficacy and safety [83-86]. The most common AEs observed with tavapadon were headache, nausea, and vomiting, with abnormal dreams and dizziness, also observed in the MAD trial [50]. AEs in the MAD trial most commonly occurred during the up-titration phase of both studies and therefore appeared to be related to dose increases rather than the maximal dose exposure [50].

The efficacy and safety of tavapadon were further investigated in a phase 2 randomized, controlled trial in individuals with early-stage PD [52]. Fifty-seven treatment-naïve patients with PD were treated with tavapadon (N=29) or placebo (N=28) over a 15-week study period (9-week dose optimization and 6-week dose maintenance) [52]. Patients treated with tavapadon showed significantly greater reductions in motor symptoms compared with patients who received a placebo, as measured by the MDS-UPDRS-III [52]. Improvements in motor control were evident following three weeks of treatment and were sustained at all test time points (6, 9, and 12 weeks) through the end of the 15-week study period [52]. The combined MDS-UPDRS score (including parts I, II, and III) and scores on the Patient Global Impression of Improvement scale were also significantly improved in patients treated with tavapadon at weeks 9 and 15 [52]. No patients in this trial required any dose adjustments during the last four weeks of the 22-week study period, suggesting tavapadon’s potential for sustained motor control [52].

In the phase 2 trial, tavapadon was generally well tolerated [52]. There were no significant differences in scores on the Beck Depression Inventory-II, the Epworth Sleepiness Scale (ESS), the Columbia Suicide Severity Rating Scale (C-SSRS), the Questionnaire for Impulsive Compulsive Disorders in Parkinson’s Disease (QUIP-RS), or the Physician Withdrawal Checklist between the cohorts of patients treated with tavapadon or placebo [52]. The most commonly reported AEs in tavapadon-treated patients included nausea (31%), headache (24%), dry mouth (17%), somnolence (14%), and tremors (14%) [52]. Two patients in the tavapadon group discontinued treatment due to AEs, compared with 4 patients receiving a placebo [52]. Tavapadon produced consistent but mild decreases in systolic and diastolic blood pressure, increases in heart rate, and modest changes in other cardiovascular parameters. Although more patients treated with tavapadon had alterations in blood pressure, there was no significant increase in hypotension-related AEs [52]. While this study suggested that tavapadon was generally well tolerated and provided motor benefit for the treatment of early-stage PD, enrollment was terminated early in this study due to its link to a concurrent phase 2 trial of tavapadon in advanced PD (NCT02687542) that failed to meet a prespecified efficacy threshold in an interim futility analysis [52].

In addition to tavapadon, another D1/D5-selective partial agonist (PF-06412562) has been previously evaluated in PD. In one trial, 13 patients with PD received a split dose of PF-06412562 or placebo following initial treatment with levodopa [87]. Although the primary endpoint of device-measured finger tapping (a marker of bradykinesia) had a variable baseline and thus did not reach the prespecified efficacy criteria, there was a significant improvement in the more established MDS-UPDRS-III score from baseline at 1.5 to 2 hours following administration with PF-06412562 *vs.* placebo [87]. A comparison of additional time points showed improvements in motor symptoms starting at this initial time point that declined over the full 12-hour study period, which paralleled the PK profile of PF-06412562 [87]. Similar to tavapadon, there were no clinically significant acute changes in cardiovascular parameters, with the most common AEs being nausea and fatigue [87]. In a separate randomized, double-blind, crossover feasibility study, six patients with advanced PD (Hoehn and Yahr Stage >IV) received PF-06412562 or levodopa for two days [88]. Here, PF-06412562 had no significant effect on ratings of Clinical Global Impression of Change provided by clinicians, although the short duration of treatment and small sample size may have limited the ability to make reliable comparisons, particularly due to the high variability in responses [88]. However, caregivers consistently rated PF-06412562 efficacy to be better than levodopa, and also reported qualitative improvements in functioning [89]. Further, PF-06412562 produced no significant differences in blood pressure or heart rate [88]. Overall, these results corroborate observations in studies of tavapadon and support the therapeutic potential of D1/D5-selective partial agonism in PD.

The results from these early-phase trials have supported further clinical investigation of tavapadon for the treatment of PD. Currently, there are four ongoing phase 3 clinical trials to further evaluate the safety and efficacy of tavapadon (Table **2**) [83-86]. TEMPO-1 (NCT04201093) and TEMPO-2 (NCT04223193) are phase 3, randomized, placebo-controlled, 27-week trials to evaluate the potential of fixed-dose tavapadon (TEMPO-1; 5 and 15 mg QD; approximate enrollment, 522 patients) or flexible-dose tavapadon (TEMPO-2; 5-15 mg QD; approximate enrollment, 296 patients) as a monotherapy for patients with early-stage PD [83, 84]. The primary efficacy endpoint is the change from baseline in the MDS-UPDRS Parts II and III combined score [83, 84]. TEMPO-3 (NCT04542499) is a phase 3, randomized, placebo-controlled, 27-week trial to further establish the efficacy of flexible-dose tavapadon (5-15 mg QD) as an adjunctive therapy to levodopa in approximately 368 patients with advanced PD [85]. For this trial, the primary outcome measurement is the change from baseline in the total “ON” time patients experience without troublesome dyskinesias [85]. Specific secondary and exploratory measures to evaluate the patient's quality of life and AEs such as ICDs and excessive sleepiness will also be assessed in these trials. Patients who complete TEMPO-1/-2/-3 may also be eligible to continue treatment as part of the open-label, 58-week TEMPO-4 trial (NCT04760769) [86]. This trial, which will also enroll new patients at US-based trial sites, will further assess the safety and efficacy of tavapadon over longer treatment periods [86].

## 4 DISCUSSION

The PD treatment landscape has an unmet need for innovative treatment options, both as monotherapies and adjunctive therapies to levodopa [3]. There are currently no approved therapies that are able to successfully modify disease progression, necessitating the use of symptomatic treatments to improve patient’s quality of life [5]. Even with the broad range of symptomatic treatment options that are currently available, patients with PD report significantly less treatment satisfaction than patients with other chronic diseases [90], and unpredictable motor fluctuations in particular are associated with reduced quality of life [91]. Patients and their physicians desire options that offer effective long-term treatment of motor and nonmotor symptoms with improved ease of administration and reduced risk of AEs [90, 92, 93]. Therefore, novel therapeutics for the treatment of PD should (1) have sustained and predictable efficacy, (2) provide strong control of motor symptoms, and (3) ameliorate significant AEs that are associated with current therapies. Tavapadon, a highly selective partial agonist for D1 and D5 receptors [52], holds promise for each of these three criteria. First, by acting on D1/D5 dopamine receptors, tavapadon selectively activates direct pathway MSNs, which may provide stronger relief of motor symptoms than D2/D3-selective DAs [27, 37]. Initial preclinical and early-phase clinical trial data suggest that tavapadon provides strong motor control in PD, though additional data from ongoing phase 3 trials are needed to confirm these findings [33, 50, 52]. Second, due to its long half-life, tavapadon allows for once-daily oral dosing that may enable more predictable daily motor control than some currently available therapies [19, 50]. The partial D1/D5 agonism provided by tavapadon may also reduce the probability of receptor desensitization, increasing the likelihood of sustained treatment benefit [20]. However, larger trials over longer study periods and confirmed evidence in other populations with PD (*e.g.* advanced PD) are needed to establish sustained benefits over time. Third, tavapadon may offer an improved safety profile over levodopa, D2/D3 DAs. The combination of extended bioavailability and partial agonism of tavapadon may reduce the risk of dyskinesias that are associated with levodopa and full DAs [20, 43, 46, 80]. Selective targeting of D1/D5 receptors may also reduce the risk of AEs associated with off-target effects of D2/D3-selective DAs, as corroborated by preclinical studies [11, 58]. The partial agonism and novel chemical structure of tavapadon may also account for the lack of observed cardiovascular AEs that have plagued previous iterations of full D1/D5-selective DAs [31, 39, 50, 51]. If ongoing phase 3 trials confirm the safety and efficacy of tavapadon as a monotherapy and adjunct to levodopa, the future availability of a D1/D5-selective agonist may provide another tool for physicians to manage symptoms and AEs for patients with PD [5, 33].

## CONCLUSION

Currently available evidence supports the development of tavapadon as a novel D1/D5 receptor-targeted therapy for PD that may provide sustained and predictable relief of motor symptoms with a reduced likelihood of AEs. Ongoing phase 3 trials will provide further evidence regarding the potential of tavapadon to address the unmet needs of patients with PD.

## Figures and Tables

**Table 1:** Summary of published findings related to the efficacy and safety of tavapadon for treatment of Parkinson’s disease.

**Authors**	**Study Population**	**Study Design**	**Key Efficacy Findings**	**Key Safety Findings**
Sohur *et al* [50]	• 18 adult males and females with idiopathic PD	• Randomized, double-blind, placebo-controlled single ascending dose (0.75, 1.5, and 3.0 mg or 3.0, 6.0, and 9.0 mg) crossover study of acute tavapadon following levodopa washout	• 9.0 mg of tavapadon improved motor control for 1-12 hours after administration • MDS-UPDRS-III tavapadon *vs.* placebo: -11.13 ± 3.68 (95% CI, -17.21 to -5.06)	• Tavapadon was safe and well tolerated
	• 50 adult males and females with idiopathic PD	• Randomized, open-label multiple ascending-dose studies of once-daily tavapadon (titrated to 5, 15, or 25 mg) over 21 days	• Once-daily 15 or 25 mg tavapadon led to sustained reductions in MDS-UPDRS-III scores on day 22 • 44% of patients treated with 5 mg, 78% of patients treated with 15 mg, and 50% of patients treated with 25 mg tavapadon experienced >10 levodopa-free days	• 172 all-causality AEs were reported; most AEs were mild or moderate in severity • 6 AEs were severe • AEs led to study discontinuation in 11 patients
Reisenberg *et al* [52]	• Adult males and females with a clinical diagnosis of Parkinson’s disease (Hoehn & Yahr Stage I-III), MDS-UPDRS-III score ≥10 • Patients were treatment naïve or had <28 days of treatment with dopaminergic agents prior to the study	• Randomized, double-blind, placebo-controlled, flexible-dose study of once-daily tavapadon over 15 weeks (9-week dose optimization phase and 6-week dose maintenance phase)	• Patients receiving tavapadon reported an improvement over placebo of 4.8 ± 2.26 (90% CI, 1.0, 8.6) in MDS-UPDRS-III scores after 15 weeks • Improvements were also observed relative to the placebo at all time points prior to week 15	• 25 patients receiving tavapadon and 18 patients receiving the placebo reported TEAEs • 1 AE was severe • 2 patients in the tavapadon group and 4 patients in the placebo group discontinued the study due to AEs • More patients in the tavapadon group reported reductions in blood pressure that did not result in hypotension-related AEs (*e.g.* dizziness)

**Note:** ^a^Percentage of maintenance dose of levodopa. **Abbreviations:** AE, adverse event; LID, levodopa-induced dyskinesia; MDS-UPDRS-III, Movement Disorder Society Unified Parkinson’s Disease Rating Scale Part III; PD, Parkinson’s disease; QTcF, QT interval corrected for heart rate by Fredericia’s formula; TEAE, treatment-emergent adverse event.

**Table 2:** Summary of ongoing phase 3 trials of tavapadon for the treatment of Parkinson’s disease.

**Trial Identifier**	**Study Design**	**Key Eligibility Criteria**	**Outcome Measures**	**Estimated Completion^a^**
TEMPO-1 (NCT04201093) [83]	• Phase 3, double-blind, parallel-group RCT in patients with early PD • 27 weeks of treatment with tavapadon (5 or 15 mg QD) or a placebo	• Diagnosis of PD for <3 years • Modified Hoehn and Yahr stage 1, 1.5, or 2 • Excludes patients with a history of essential tremors, atypical or secondary parkinsonian syndrome, impulse control disorder, hallucinations, or significant neurological disorder • Excludes patients with a history of nonresponse or insufficient response to levodopa	• Primary: Change from baseline in MDS-UPDRS Parts II and III combined score • Secondary: PGI-C, MDS-UPDRS Parts I, II, and III combined and individual scores, CGI-S, CGI-I, ESS, QUIP-RS, C-SSRS, TEAEs	• Primary completion: September 2023 • Study completion: October 2023
TEMPO-2 (NCT04223193) [84]	• Phase 3, double-blind, parallel-group, flexible-dose RCT in patients with early PD • 27 weeks of treatment with tavapadon (titrated to 15 mg QD) or a placebo	• Diagnosis of PD for <3 years • Modified Hoehn and Yahr stage 1, 1.5, or 2 • Excludes patients with a history of essential tremors, atypical or secondary parkinsonian syndrome, impulse control disorder, hallucinations, or significant neurological disorder • Excludes patients with a history of nonresponse or insufficient response to levodopa	• Primary: Change from baseline in MDS-UPDRS Parts II and III combined score • Secondary: PGI-C, MDS-UPDRS Parts I, II, and III combined and individual scores, CGI-S, CGI-I, ESS, QUIP-RS, C-SSRS, TEAEs	• Primary completion: July 2023 • Study Completion: August 2023
TEMPO-3 (NCT04542499) [85]	• Phase 3, double-blind, parallel-group, flexible-dose RCT in levodopa-treated adults with motor fluctuations • 27 weeks of treatment with tavapadon (titrated to 15 mg QD) or a placebo	• Diagnosis of PD • Modified Hoehn & Yahr stage 2, 2.5, or 3 in the “ON” state • Good response to levodopa and stable dose of at least 400 mg for 4 weeks prior to screening • At least 2.5 hours of daily “OFF” time • Excludes patients with a history of essential tremors, atypical or secondary parkinsonian syndrome, impulse control disorder, hallucinations, or significant neurological disorder • Excludes patients with a history of nonresponse or insufficient response to levodopa	• Primary: Change from baseline in total “ON” time without troublesome dyskinesia at 27 weeks • Secondary: Change from baseline in total “OFF” time at 27 weeks and other time points, change from baseline in total “ON” time without troublesome dyskinesia at other time points, change from baseline in MDS-UPDRS Parts I, II, and III individual scores	• Primary completion: February 2023 • Study completion: March 2023
TEMPO-4 (NCT04760769) [86]	• Phase 3, open-label extension trial • 58 weeks of treatment with tavapadon (5-15 mg QD)	• Prior enrollment in and completion of another TEMPO trial OR • Diagnosis of PD, modified Hoehn and Yahr stage 1, 1.5, 2, 2.5 or 3 • Currently treated with levodopa/ carbidopa • Excludes patients with a history of essential tremor, atypical or secondary parkinsonian syndrome, impulse control disorder, hallucinations, or significant neurological disorder • Excludes patients with a history of nonresponse or insufficient response to levodopa	• TEAEs, treatment discontinuation • QUIP-RS, ESS, C-SSRS, SMWQ • Change from baseline in MDS-UPDRS parts I, II, and III, Hauser diary, EQ-5D-5L index, and visual analog scores	• Primary completion: November 2024 • Study completion: December 2024

^a^Per ClinicalTrials.gov as of July 2022.

**Abbreviations**: CGI-I, Clinical Global Impression-Improvement; CGI-S, Clinical Global Impression-Severity; C-SSRS, Columbia-Suicide Severity Rating Scale; ESS, Epworth Sleepiness Scale; EQ-5D-5L, EuroQol 5 Dimension 5 Level; MDS-UPDRS, Movement Disorder Society - Unified Parkinson’s Disease Rating Scale; PD, Parkinson’s disease; PGI-C, Patient Global Impression-Change; QUIP-RS, Questionnaire for Impulsive-Compulsive Disorders in Parkinson’s Disease-Rating Scale; RCT, randomized controlled trial; SMWQ, Study Medication Withdrawal Questionnaire; TEAE, treatment-emergent adverse event.

## LIST OF ABBREVIATIONS

AAN: American Academy of Neurology
AE: Adverse Event
C-SSRS: Columbia Suicide Severity Rating Scale
COMT: Catechol-O-methyl Transferase
DA: Dopamine Agonist
EC_50_: Half-maximal Effective Concentration
EDS: Excessive Daytime Sleepiness
ESS: Epworth Sleepiness Scale
ICD: Impulse Control Disorder
MAD: Multiple Ascending Doses
MDS-UPDRS-III: Movement Disorder Society-Unified Parkinson’s Disease Rating Scale Part III
MPTP: 1-methyl-4-phenyl-1,2,3,6-tetrahydropyridine
MSN: Medium Spiny Neuron
NDMA: N-methyl-D-aspartate
PD: Parkinson’s Disease
PK: Pharmacokinetic
QD: Once Daily
QUIP-RS: Questionnaire for Impulsive Compulsive Disorders in Parkinson’s Disease
SAD: Single Ascending Dose
